# High neutrophil-to-albumin ratio signals severe prognosis in LVO stroke—data from an EICU cohort

**DOI:** 10.3389/fmed.2026.1753227

**Published:** 2026-04-01

**Authors:** Chao Yang, Jingying Wang, Yiyao Lu, Yinghe Xu, Yongpo Jiang, Hui Zhao

**Affiliations:** 1 Department of Emergency Medicine, Taizhou Hospital of Zhejiang Province Affiliated with Wenzhou Medical University, Taizhou, China; 2 Department of Critical Care Medicine, Taizhou Hospital of Zhejiang Province Affiliated with Wenzhou Medical University, Taizhou, China

**Keywords:** inflammation, intracerebral hemorrhage, mechanical thrombectomy, neutrophil-to-albumin ratio, prognosis, stroke

## Abstract

**Objective:**

To investigate whether the neutrophil-to-albumin ratio (NPAR) predicts adverse outcomes in acute large vessel occlusion (LVO) stroke.

**Methods:**

This cohort study enrolled 893 LVO stroke patients (2021–2023). Associations between NPAR and outcomes were assessed using logistic regression, restricted cubic spline, and ROC analysis.

**Results:**

Higher NPAR correlated with elevated NIHSS, inflammatory markers, coagulation parameters, and dysphagia, and decreased hemoglobin, lymphocytes, and GCS. NPAR was an independent predictor for 90-day poor functional outcome (OR = 1.10, 95%CI: 1.07–1.14), pneumonia (OR = 1.14, 95%CI: 1.10–1.18), and intracerebral hemorrhage (ICH) (OR = 1.06, 95%CI:1.03–1.10). After multivariable adjustment, associations remained robust (aOR: 1.06–1.14). Linear dose–response relationships were observed. NPAR showed modest predictive performance (AUC: 0.60–0.65). Adding NPAR significantly improved pneumonia prediction over AIS-APS (AUC: 0.684 → 0.709, *p* = 0.0049), enhanced ICH sensitivity (56.6% → 64.6%), but minimally improved 90-day outcome prediction.

**Conclusion:**

Elevated NPAR independently predicts pneumonia, ICH, and poor functional recovery in LVO stroke, serving as a potential early risk stratification tool. However, these findings should be interpreted in light of the study’s limitations, including its single-center retrospective design, single time-point NPAR measurement, and predominantly Han Chinese population, which may limit generalizability.

## Introduction

Stroke is the second most common cause of death worldwide, accounting for approximately 7 million fatalities each year, and it ranks third in terms of combined mortality and disability (1). It is responsible for approximately 11% of global mortality (2). Although advances in treatment modalities, such as thrombolysis and thrombectomy, have contributed to a reduction in disability and mortality rates (3), the rate of long-term disability remains as high as approximately 50% in stroke patients with large vessel occlusion (LVO) (4). The modified Rankin scale (mRS) is widely recognized as a key indicator for assessing outcomes in acute ischemic stroke (AIS) patients and directly reflects patients’ functional independence and mortality (5). As two of the most common complications of stroke, intracerebral hemorrhage (ICH) and stroke-associated pneumonia (SAP) are associated not only with prolonged hospital stays and increased economic burden but also strongly correlated with functional independence and mortality in stroke patients (6–9). Our findings highlight the need for a simple and effective biomarker to predict overall stroke prognosis and guide earlier clinical intervention.

The NPAR, an emerging clinical biomarker, is a readily accessible measure of systemic inflammation due to the ease of data collection and calculation from routine blood tests (10). It has shown promising prognostic value in various conditions, including cardiovascular diseases (11, 12), cerebrovascular diseases (10, 13), renal injury (14), and cancer-related conditions (15). The mechanisms involve neutrophil-driven inflammation (16) and the promotion of thrombosis (17), which are critical for exacerbating ischemic injury and worsening stroke outcomes. Conversely, albumin is considered a neuroprotective agent in acute stroke (18). The composite marker NPAR may thus reflect a balance between the inflammatory state, thrombotic tendency, and impaired antioxidant defense (10), potentially aiding in the clinical assessment of the progression and outcomes of acute stroke.

Despite the established burden of LVO stroke, the prognostic value of the NPAR within this population has seldom been examined. We address this gap by evaluating how NPAR stratification aligns with the clinical phenotype and by quantifying its association with three key complications: poor functional independence, pneumonia, and intracerebral hemorrhage.

## Methods

### Participants

**Figure 1:**
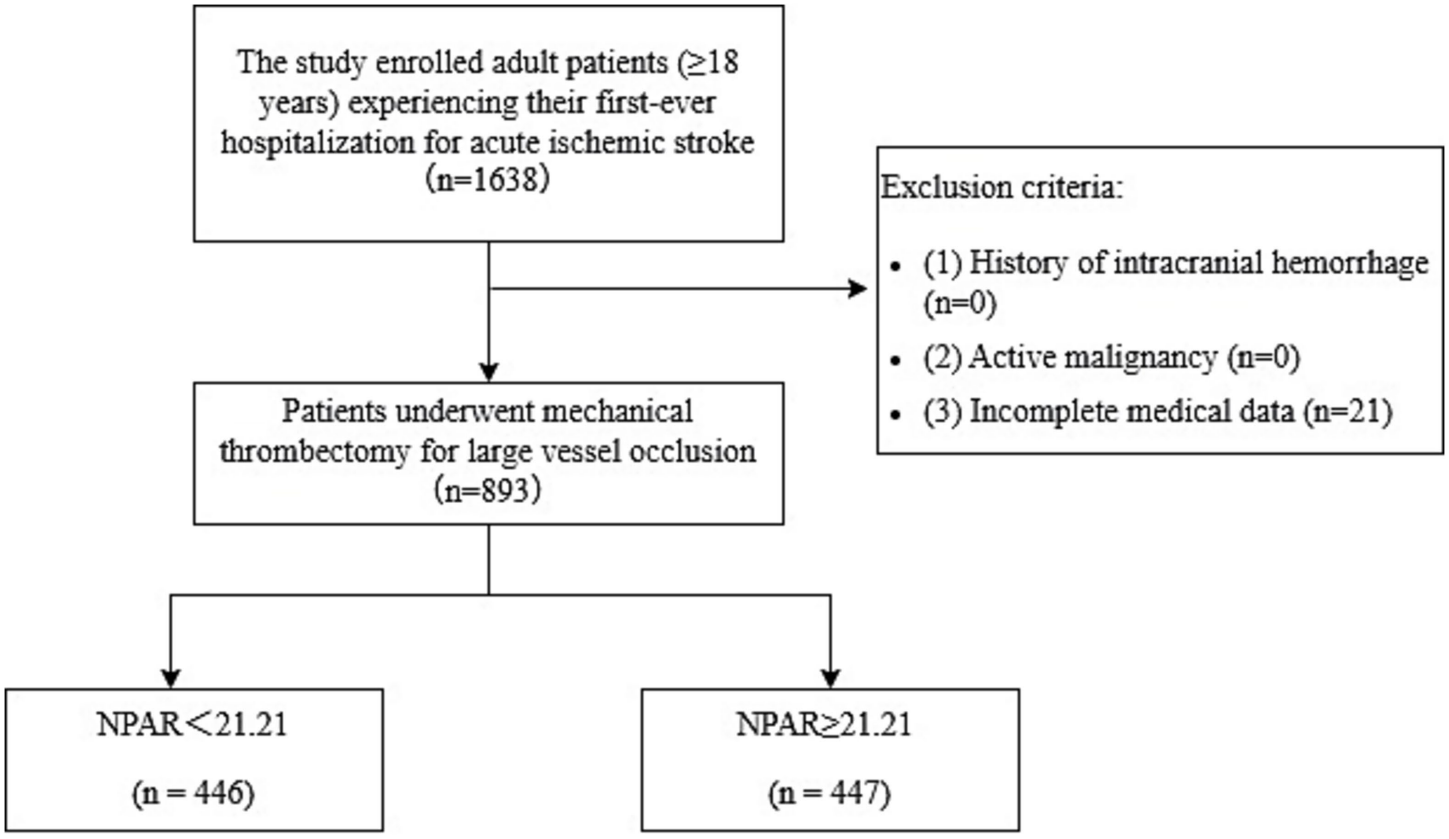
Study flow diagram.

Between January 2021 and August 2023, all adults (≥ 18 y) admitted to Taizhou Enze Medical Center (Zhejiang, China) with acute LVO documented by digital subtraction angiography and treated with emergency mechanical thrombectomy were retrospectively reviewed. Postprocedural care was delivered in the Emergency Intensive Care Unit. The Institutional Ethics Committee (approval K20240505) exempted informed consent owing to the retrospective, anonymized nature of the analysis. Patients were excluded if they had antecedent intracranial hemorrhage, active malignancy, or insufficient records. Specifically, 21 patients were excluded due to incomplete medical records, ensuring that all remaining 893 patients had complete data for all variables included in the analysis. The patient selection process followed a predefined algorithm (see Figure 1).

### Study procedure

From electronic charts, we extracted three domains of information. First, the baseline profiles included sex, age, onset-to-treatment time, vascular risk factors (hypertension, diabetes mellitus, and atrial fibrillation), admission vital signs, GCS score, NIHSS score, ASPECTS, and occluded vessel territory. Second, periprocedural indices, i.e., preoperative and day-1 laboratory values—white blood cell, neutrophil, lymphocyte, and platelet counts; hemoglobin, CRP, PCT, triglyceride, INR, APTT, fibrinogen, thrombin time, D-dimer, and serum albumin—together with mechanical ventilation requirements and dysphagia status, were measured. The NPAR was computed as the neutrophil percentage (%) divided by the albumin concentration (g dL^−1^) from the first paired sample drawn after EICU admission following mechanical thrombectomy (MT) and patient transfer to the intensive care unit. Third, 90-day functional outcomes (modified Rankin scale score ≥ 3 indicated poor recovery), in-hospital pneumonia, and any radiologically confirmed intracerebral hemorrhage were recorded as primary endpoints.

### Statistical methods

All computations were performed in SPSS 27.0 (IBM, Armonk, NY) and R 4.3.2 (R Foundation, Vienna). Patients were split at the cohort-specific NPAR median (21.21) into “low” and “high” groups for descriptive comparison. Continuous variables were first examined with the Shapiro–Wilk test; normally distributed metrics are reported as the means ± SDs and were compared with two-sample t tests, whereas skewed data are described as medians (IQRs) and were analyzed with the Mann–Whitney U test. Count data are expressed as *n* (%) and were evaluated with the *χ*^2^ test or Fisher’s exact test as appropriate.

Missing data were addressed at the patient selection stage. A total of 21 patients were excluded from the initial cohort due to incomplete medical records (as detailed in the Participants section and Figure 1). Consequently, the final analytical sample comprised 893 patients with complete data for all variables. No additional imputation methods (such as multiple imputation or complete case analysis within the analytical stage) were required.

To identify candidate predictors of each endpoint, univariable logistic regression was applied. Multivariable logistic regression was used to assess the independent association of NPAR with outcomes, adjusting for age, NIHSS, glucose, and onset-to-treatment time. Subgroup analyses were performed stratified by age (<70 vs. ≥70 years), sex, hypertension, and atrial fibrillation, with interaction terms tested to evaluate effect consistency across subgroups. The shape of the NPAR–outcome relationship was visualized with restricted cubic splines (four knots at fixed percentiles). Discrimination of the NPAR was quantified by the area under the receiver operating characteristic curve (AUC), and its value was contrasted with that of established scores (NIHSS, GCS, ASPECTS) via the DeLong method. To assess potential multicollinearity among predictors, variance inflation factors (VIFs) were calculated for variables included in the regression models. A VIF value > 5 was considered indicative of concerning collinearity, and > 10 indicated serious multicollinearity. Given that the primary analyses were univariable, multicollinearity was not a concern in the main logistic regression models; however, this assessment was performed to inform any sensitivity analyses and to ensure the robustness of the findings. All tests were two-tailed, with *p* < 0.05 considered significant.

## Results

### Participant characteristics

Among the 893 individuals included in the analysis, 549 (61.5%) were men, and the median age was 70.0 years (IQR 60–77). After the cohort at the NPAR median (21.21) was split, the high-NPAR stratum was older and presented a higher rate of atrial fibrillation than did the low-NPAR stratum; all other baseline variables were comparable between the groups (Table 1).

**Table 1:** Associations between the NPAR and baseline characteristics of the participants.

Variables	Total (*n* = 893)	NPAR<21.21 (*n* = 446)	NPAR≥21.21 (*n* = 447)	Statistic	*p*
Age, M (Q₁, Q₃)	70.00 (60.00, 77.00)	67.00 (57.00, 74.75)	71.00 (64.00, 79.00)	*Z* = −5.51	<0.001
Time of onset, M (Q₁, Q₃)	5.00 (3.00, 8.00)	4.50 (3.00, 9.00)	5.00 (3.00, 7.50)	*Z* = −0.10	0.920
Sex, *n* (%)				*χ*^2^ = 0.64	0.424
Female	344 (38.52)	166 (37.22)	178 (39.82)		
Male	549 (61.48)	280 (62.78)	269 (60.18)		
Hypertension, *n* (%)				*χ*^2^ = 0.43	0.510
No	348 (38.97)	169 (37.89)	179 (40.04)		
Yes	545 (61.03)	277 (62.11)	268 (59.96)		
Diabetes, *n* (%)				*χ*^2^ = 0.45	0.502
No	703 (78.72)	347 (77.80)	356 (79.64)		
Yes	190 (21.28)	99 (22.20)	91 (20.36)		
Smoke, *n* (%)				*χ*^2^ = 2.61	0.106
No	658 (73.68)	318 (71.30)	340 (76.06)		
Yes	235 (26.32)	128 (28.70)	107 (23.94)		
COPD, *n* (%)				*χ*^2^ = 1.66	0.197
No	871 (97.54)	438 (98.21)	433 (96.87)		
Yes	22 (2.46)	8 (1.79)	14 (3.13)		
History of cerebral infarction, *n* (%)				*χ*^2^ = 0.57	0.449
No	726 (81.30)	367 (82.29)	359 (80.31)		
Yes	167 (18.70)	79 (17.71)	88 (19.69)		
Atrial fibrillation, *n* (%)				*χ*^2^ = 19.72	<0.001
No	641 (71.78)	350 (78.48)	291 (65.10)		
Yes	252 (28.22)	96 (21.52)	156 (34.90)		
Heart failure, *n* (%)				*χ*^2^ = 2.81	0.094
No	848 (94.96)	429 (96.19)	419 (93.74)		
Yes	45 (5.04)	17 (3.81)	28 (6.26)		
Cardiac infarction, *n* (%)				*χ*^2^ = 2.60	0.107
No	879 (98.43)	442 (99.10)	437 (97.76)		
Yes	14 (1.57)	4 (0.90)	10 (2.24)		
Thrombolysis, *n* (%)				*χ*^2^ = 0.69	0.407
No	668 (74.80)	339 (76.01)	329 (73.60)		
Yes	225 (25.20)	107 (23.99)	118 (26.40)		
Anterior circulation infarction, *n* (%)				*χ*^2^ = 0.51	0.476
No	144 (16.13)	68 (15.25)	76 (17.00)		
Yes	749 (83.87)	378 (84.75)	371 (83.00)		

Z, Mann–Whitney test; *χ*^2^, Chi-square test.

M, Median; Q₁, 1st Quartile; Q₃, 3 st Quartile.

### Comparison of clinical and laboratory covariates

A comprehensive comparison of the clinical and laboratory parameters between the high- and low-NPAR groups is summarized in Table 2. Clinically, a higher NPAR was associated with a lower initial Glasgow Coma Scale (GCS) score [median (IQR): 11.00 (8.00, 12.00) vs. 12.00 (10.00, 12.00)] and a higher National Institutes of Health Stroke Scale (NIHSS) score [15.00 (11.00, 19.00) vs. 11.00 (6.00, 16.00)]. Furthermore, the proportion of patients who experienced a GCS score decrease of more than 3 points within 72 h was significantly greater in the high NPAR group (65 patients, 14.54%) than in the low NPAR group (32 patients, 7.17%).

**Table 2:** Distribution of characteristics in the NPAR group by covariates.

Variables	Total (*n* = 893)	NPAR<21.21 (*n* = 446)	NPAR≥21.21 (*n* = 447)	Statistic	*p*
Admission temperature, M (Q₁, Q₃)	36.60 (36.50, 36.80)	36.60 (36.50, 36.80)	36.60 (36.50, 36.80)	*Z* = −0.42	0.675
Admission blood pressure, M (Q₁, Q₃)	150.00 (134.00, 167.00)	149.50 (133.00, 165.00)	150.00 (135.00, 169.50)	*Z* = −0.57	0.566
Admission GCS, M (Q₁, Q₃)	11.00 (9.00, 12.00)	12.00 (10.00, 12.00)	11.00 (8.00, 12.00)	*Z* = −6.24	<0.001
NIHSS, M (Q₁, Q₃)	13.00 (8.00, 18.00)	11.00 (6.00, 16.00)	15.00 (11.00, 19.00)	*Z* = −8.06	<0.001
WBC, M (Q₁, Q₃)	8.20 (6.60, 10.60)	8.00 (6.53, 9.90)	8.50 (6.65, 10.90)	*Z* = −2.54	0.011
HB, M (Q₁, Q₃)	136.00 (124.00, 148.00)	140.00 (129.25, 150.00)	131.00 (119.00, 145.00)	*Z* = −6.70	<0.001
PLT, M (Q₁, Q₃)	204.00 (166.00, 248.00)	210.00 (176.00, 248.75)	197.00 (156.00, 244.00)	*Z* = −2.91	0.004
MonoC, M (Q₁, Q₃)	0.40 (0.30, 0.60)	0.40 (0.30, 0.60)	0.40 (0.30, 0.60)	*Z* = −2.00	0.046
Lymph, M (Q₁, Q₃)	1.40 (1.00, 2.00)	1.60 (1.20, 2.20)	1.30 (0.90, 1.80)	*Z* = −7.09	<0.001
PostWBC, M (Q₁, Q₃)	8.90 (7.10, 10.90)	8.40 (6.73, 10.07)	9.40 (7.55, 11.40)	*Z* = −5.52	<0.001
PostHB, M (Q₁, Q₃)	124.00 (113.00, 136.00)	128.00 (117.00, 137.00)	120.00 (109.00, 132.50)	*Z* = −6.03	<0.001
PostPLT, M (Q₁, Q₃)	194.00 (157.00, 232.00)	197.00 (164.25, 235.00)	188.00 (148.00, 230.50)	*Z* = −2.88	0.004
PostMono, M (Q₁, Q₃)	0.40 (0.30, 0.60)	0.50 (0.30, 0.60)	0.40 (0.30, 0.60)	*Z* = −1.70	0.089
PostLymph, M (Q₁, Q₃)	1.10 (0.80, 1.60)	1.30 (1.00, 1.80)	0.90 (0.65, 1.30)	*Z* = −10.10	<0.001
GLU, M (Q₁, Q₃)	7.23 (6.27, 9.23)	7.08 (6.04, 9.00)	7.42 (6.49, 9.60)	*Z* = −3.04	0.002
TG, M (Q₁, Q₃)	1.06 (0.77, 1.49)	1.13 (0.83, 1.66)	0.94 (0.72, 1.33)	*Z* = −4.86	<0.001
Cr, Mean ± SD	74.59 ± 36.31	74.92 ± 26.57	74.25 ± 43.96	*t* = 0.27	0.783
CRP, M (Q₁, Q₃)	3.30 (1.40, 8.50)	2.80 (1.10, 6.07)	4.00 (1.70, 10.75)	*Z* = −4.27	<0.001
72 h CRP Max, M (Q₁, Q₃)	24.00 (7.20, 67.70)	14.55 (5.73, 50.53)	35.80 (9.95, 101.30)	*Z* = −6.74	<0.001
PCT, M (Q₁, Q₃)	0.05 (0.03, 0.11)	0.05 (0.03, 0.08)	0.06 (0.03, 0.15)	*Z* = −3.41	<0.001
InCL, M (Q₁, Q₃)	104.70 (102.20, 107.00)	104.25 (102.00, 106.50)	105.00 (102.70, 107.10)	*Z* = −2.66	0.008
InNa, M (Q₁, Q₃)	138.60 (136.70, 140.40)	138.70 (137.30, 140.40)	138.60 (136.10, 140.50)	*Z* = −2.32	0.021
Pre PT, M (Q₁, Q₃)	13.20 (12.60, 13.70)	12.90 (12.50, 13.43)	13.40 (12.80, 14.10)	*Z* = −6.97	<0.001
Pre INR, M (Q₁, Q₃)	1.02 (0.96, 1.08)	1.00 (0.95, 1.05)	1.04 (0.98, 1.11)	*Z* = −7.05	<0.001
Pre APTT, M (Q₁, Q₃)	33.90 (31.10, 37.20)	33.65 (30.80, 36.50)	34.30 (31.40, 38.10)	*Z* = −2.94	0.003
Pre Fib, M (Q₁, Q₃)	3.19 (2.72, 3.76)	3.16 (2.74, 3.69)	3.22 (2.70, 3.88)	*Z* = −1.13	0.260
TT, M (Q₁, Q₃)	17.80 (16.90, 18.90)	17.70 (16.90, 18.70)	17.90 (17.00, 19.30)	*Z* = −3.00	0.003
DDimer, M (Q₁, Q₃)	1.25 (0.62, 2.38)	0.96 (0.51, 1.89)	1.55 (0.81, 2.60)	*Z* = −6.61	<0.001
Post PT, M (Q₁, Q₃)	13.70 (13.20, 14.30)	13.50 (13.00, 14.00)	13.80 (13.30, 14.55)	*Z* = −5.52	<0.001
Post INR, M (Q₁, Q₃)	1.07 (1.02, 1.13)	1.05 (1.00, 1.11)	1.09 (1.03, 1.16)	*Z* = −5.61	<0.001
Post APTT, M (Q₁, Q₃)	35.10 (32.70, 38.30)	35.00 (32.80, 37.20)	35.40 (32.70, 38.90)	*Z* = −1.56	0.118
Post fib, M (Q₁, Q₃)	3.15 (2.69, 3.73)	3.11 (2.65, 3.57)	3.19 (2.73, 3.81)	*Z* = −2.58	0.010
Post TT, M (Q₁, Q₃)	17.30 (16.50, 18.50)	17.50 (16.60, 18.70)	17.20 (16.40, 18.40)	*Z* = −2.00	0.045
DownGCS≥3 sore, *n* (%)				*χ*^2^ = 12.51	<0.001
No	796 (89.14)	414 (92.83)	382 (85.46)		
Yes	97 (10.86)	32 (7.17)	65 (14.54)		
Dysphagia, *n* (%)				*χ*^2^ = 42.02	<0.001
No	406 (45.46)	251 (56.28)	155 (34.68)		
Yes	487 (54.54)	195 (43.72)	292 (65.32)		
Mechanical ventilation, *n* (%)				*χ*^2^ = 0.20	0.653
No	677 (75.81)	341 (76.46)	336 (75.17)		
Yes	216 (24.19)	105 (23.54)	111 (24.83)		

*t*, *t*-test; Z, Mann–Whitney test; *χ*^2^, Chi-square test.

SD, standard deviation; M, Median; Q₁, 1st Quartile; Q₃, 3 st Quartile.

In terms of laboratory findings, elevated NPAR was consistently associated with higher white blood cell counts and lower levels of hemoglobin, lymphocytes, and platelets, both preoperatively and on the first postoperative day. Marked differences were observed in the levels of inflammatory markers. The maximum C-reactive protein (CRP) level within 72 h was significantly greater in the high NPAR group [35.80 mg/L (9.95, 101.30)] than in the low NPAR group [14.55 mg/L (5.73, 50.53)], a finding that was notable despite the absence of significant differences in body temperature upon admission. Significant disparities were also evident in electrolyte levels (chloride, *p* = 0.008; sodium, *p* = 0.021), blood glucose, and triglyceride levels. Interestingly, triglyceride levels were lower in the high NPAR group [0.94 mmol/L (0.72, 1.33)] than in the low NPAR group [1.13 mmol/L (0.83, 1.66)].

The coagulation profiles also differed significantly. The high NPAR group presented a prolonged prothrombin time (PT), international normalized ratio (INR), and activated partial thromboplastin time (APTT), along with notably higher D-dimer levels [1.55 μg/mL (0.81, 2.60) vs. 0.96 μg/mL (0.51, 1.89)]. Finally, the incidence of swallowing dysfunction was substantially greater in the high NPAR group (336 patients, 75.17%) than in the low NPAR group (195 patients, 43.72%), whereas the rate of mechanical ventilation requirement did not differ significantly between the groups.

### Associations between the NPAR and clinical outcomes

**Figure 2:**
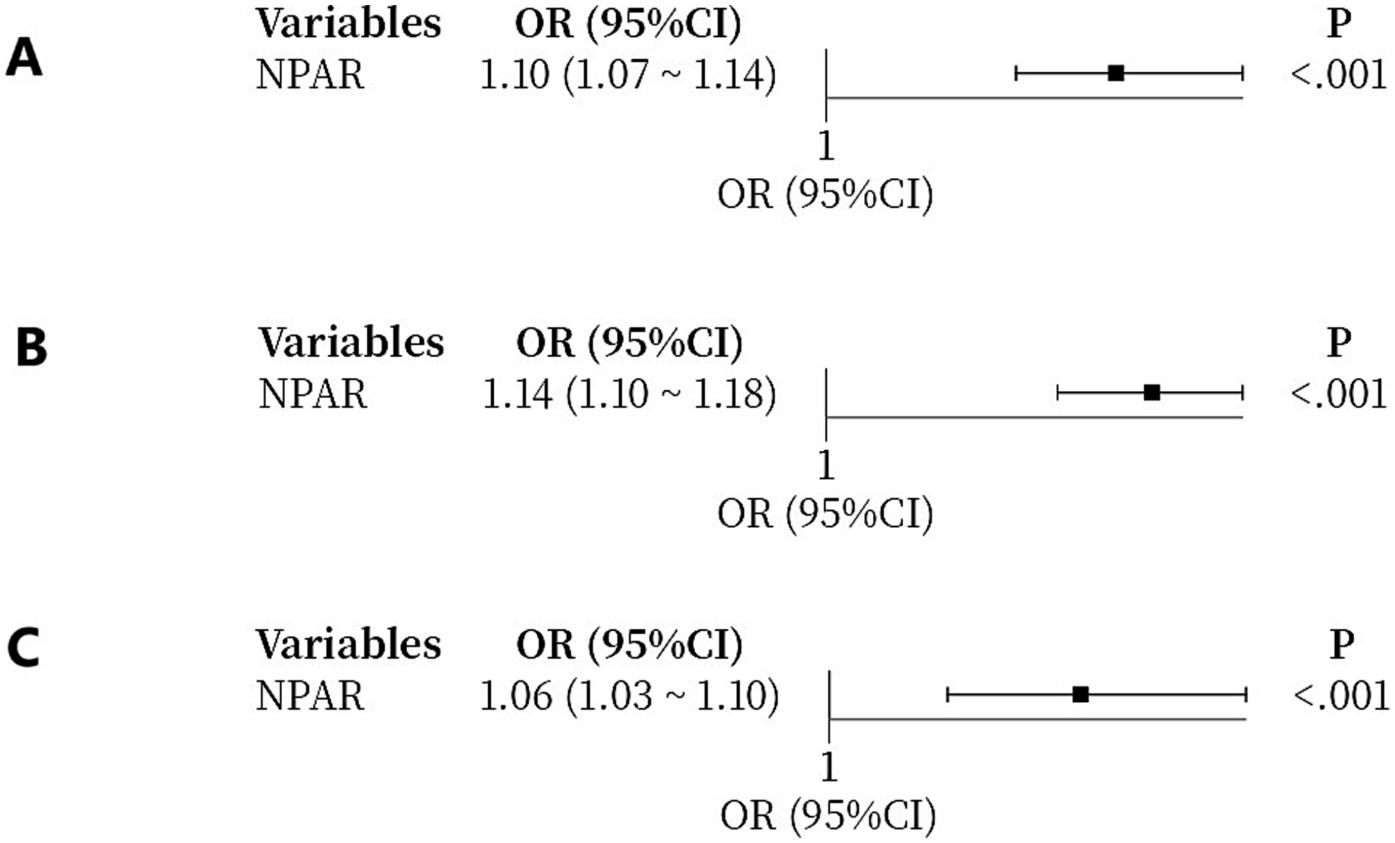
NPAR as an independent risk factor for adverse outcomes. Forest plots for **(A)** poor 90-day functional outcome (mRS ≥ 3), **(B)** pneumonia, and **(C)** intracerebral hemorrhage.

Univariable logistic models linked each unit increase in the NPAR to increased odds of an unfavorable 90-day outcome (OR 1.10, 95% CI 1.07–1.14), pneumonia (OR 1.14, 95% CI 1.10–1.18), and intracerebral hemorrhage (OR 1.06, 95% CI 1.03–1.10) (Figure 2).

After multivariable adjustment for potential confounders, including age, baseline NIHSS score, admission glucose, and onset-to-treatment time (OTT), NPAR remained significantly associated with all three adverse outcomes (Table 3). The adjusted odds ratios were 1.09 (95% CI: 1.06–1.13, *p* < 0.001) for poor 90-day functional outcome, 1.14 (95% CI: 1.10–1.18, *p* < 0.001) for pneumonia, and 1.06 (95% CI: 1.02–1.09, *p* = 0.003) for intracerebral hemorrhage. These findings confirm that NPAR is an independent predictor of adverse outcomes in LVO stroke patients undergoing mechanical thrombectomy, even after accounting for established prognostic factors.

**Table 3:** The multivariable analysis confirmed that NPAR remains significantly associated with all three adverse outcomes after adjustment:

Outcome	Adjusted ORa (95% CI)	*p* value
Poor 90-day functional outcome (mRS ≥ 3)	1.09 (1.06–1.13)	< 0.001
Pneumonia	1.14 (1.10–1.18)	< 0.001
Intracerebral hemorrhage	1.06 (1.02–1.09)	0.003

*aOR adjusted for age, baseline NIHSS score, admission glucose, and onset-to-treatment time (OTT).

Multicollinearity assessment revealed that all variance inflation factor (VIF) values were below 2.5 for the variables examined, indicating no concerning collinearity among predictors. This finding supports the stability and reliability of the regression estimates.

Subgroup analyses confirmed the robustness of these findings (Supplementary Table 1). NPAR remained significantly associated with all three outcomes across subgroups stratified by age (<70 vs. ≥70 years), sex, hypertension, and atrial fibrillation (all *p* < 0.05, except for ICH in hypertensive patients, where *p* = 0.061). No significant interactions were observed (all P for interaction > 0.05), indicating that the prognostic value of NPAR is consistent regardless of these clinical characteristics.

**Figure 3:**
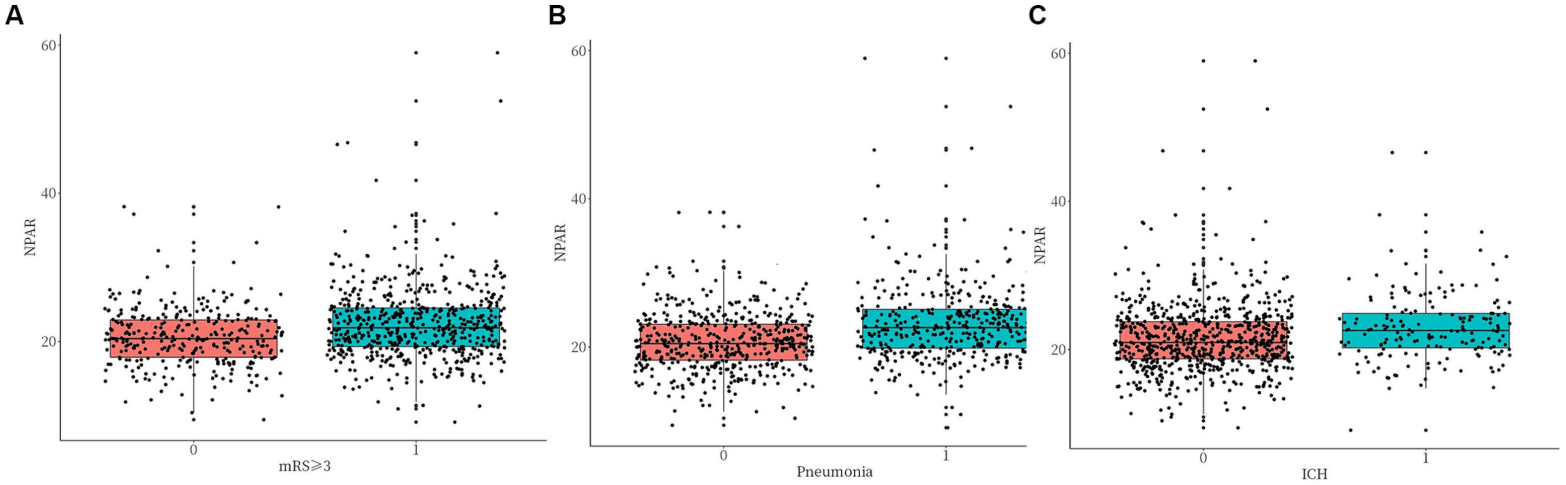
Calibration of the predicted probabilities. P–P plots comparing the predicted versus observed probabilities for **(A)** poor functional outcome (mRS ≥ 3), **(B)** pneumonia, and **(C)** ICH.

To assess the model fit and distribution of the predicted probabilities, box plots were generated (Figure 3), which indicated a consistent association between higher NPAR levels and an increased risk of all three adverse outcomes.

**Figure 4:**
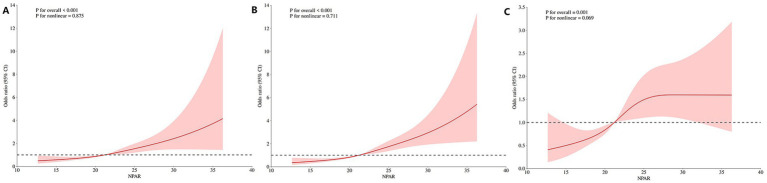
Linear dose–response relationships between the NPAR and adverse outcomes. Restricted cubic spline analyses for **(A)** 90-day mRS score ≥3, **(B)** pneumonia, and **(C)** ICH.

Furthermore, restricted cubic spline (RCS) analysis was employed to examine the shape of the dose–response relationship between the NPAR and each outcome. The results, displayed in Figure 4, revealed a near-linear association: as the NPAR increased, the risks of poor functional outcome, pneumonia, and intracerebral hemorrhage increased progressively, with no evidence of a threshold or plateau effect within the observed range.

### Predictive performance of the NPAR

To further evaluate the ability of the NPAR to predict adverse outcomes, we compared its performance with that of several commonly used neurological assessment scales; the results are detailed in Tables 4–6.

**Table 4:** AUC and Predictive Performance of the NPAR for an Unfavorable Outcome (mRS ≥ 3) at 90 Days.

Variable	AUC (95%CI)	Accuracy (95%CI)	Sensitivity (95%CI)	Specificity (95%CI)	PPV (95%CI)	NPV (95%CI)
NPAR	0.61 (0.58–0.65)	0.58 (0.54–0.61)	0.64 (0.59–0.70)	0.54 (0.50–0.58)	0.44 (0.39–0.48)	0.73 (0.69–0.77)
NIHSS	0.70 (0.66–0.73)	0.64 (0.61–0.68)	0.69 (0.64–0.74)	0.62 (0.58–0.66)	0.50 (0.45–0.55)	0.78 (0.74–0.82)
GCS	0.65 (0.62–0.69)	0.41 (0.37–0.44)	0.20 (0.16–0.24)	0.52 (0.48–0.56)	0.19 (0.15–0.23)	0.54 (0.50–0.58)
Aspects	0.57 (0.53–0.61)					

**Table 5:** Ability of the NPAR to predict pneumonia.

Variable	AUC (95%CI)	Accuracy (95%CI)	Sensitivity (95%CI)	Specificity (95%CI)	PPV (95%CI)	NPV (95%CI)
NPAR	0.60 (0.56–0.65)	0.54 (0.50–0.57)	0.50 (0.47–0.54)	0.67 (0.60–0.74)	0.86 (0.83–0.90)	0.25 (0.21–0.29)
NIHSS	0.58 (0.53–0.63)	0.47 (0.44–0.51)	0.40 (0.36–0.43)	0.78 (0.72–0.84)	0.88 (0.84–0.92)	0.24 (0.20–0.27)
GCS	0.47 (0.42–0.51)	0.20 (0.18–0.23)	0.01 (0.00–0.02)	1.00 (1.00–1.00)	1.00 (1.00–1.00)	0.20 (0.17–0.22)
Aspects	0.64 (0.60–0.69)	0.33 (0.30–0.37)	0.31 (0.27–0.34)	0.44 (0.37–0.51)	0.69 (0.64–0.74)	0.13 (0.11–0.16)

**Table 6:** Diagnostic performance of the NPAR for intracerebral hemorrhage.

Variable	AUC (95%CI)	Accuracy (95%CI)	Sensitivity (95%CI)	Specificity (95%CI)	PPV (95%CI)	NPV (95%CI)
NPAR	0.65 (0.61–0.69)	0.63 (0.59–0.66)	0.64 (0.60–0.69)	0.60 (0.56–0.65)	0.67 (0.62–0.71)	0.58 (0.53–0.63)
NIHSS	0.69 (0.66–0.73)	0.64 (0.61–0.67)	0.55 (0.51–0.59)	0.75 (0.71–0.79)	0.73 (0.69–0.78)	0.57 (0.53–0.62)
GCS	0.64 (0.60–0.67)	0.37 (0.33–0.40)	0.27 (0.23–0.31)	0.48 (0.43–0.53)	0.39 (0.34–0.44)	0.35 (0.31–0.39)
Aspects	0.45 (0.41–0.48)	0.45 (0.42–0.48)	0.00 (<0.001–0.01)	1.00 (0.99–1.00)	0.67 (0.13–1.00)	0.45 (0.42–0.48)

The discriminative ability of the NPAR for the three endpoints was modest. For poor functional recovery at 90 days, the AUC was 0.61 (95% CI 0.58–0.65), the sensitivity was 64%, the PPV was 44%, and the NPV was 73%. Pneumonia prediction was only marginally better: AUC 0.65 (0.61–0.69), sensitivity 64%, PPV 67%, NPV 58%. For intracerebral hemorrhage, the AUC reached 0.60 (0.56–0.65), with 50% sensitivity, 86% PPV, and 25% NPV.

Although the predictive performance of the NPAR for any single outcome, as indicated by the AUC, was lower than that of some established neurological scores, it demonstrated consistent and stable discriminative ability across all three adverse outcomes.

Adding NPAR to clinical scores improved prediction for all outcomes (Table 7). For pneumonia, NPAR significantly enhanced AIS-APS (AUC: 0.684 → 0.709, *p* = 0.0049), with sensitivity rising from 60.3 to 69.0%. For ICH, NPAR improved ASIAN score sensitivity from 56.6 to 64.6%, though the AUC increase (0.649 → 0.665) was not significant (*p* = 0.11). For the 90-day outcome, NPAR minimally improved PRE score (AUC: 0.694 → 0.701, *p* = 0.23), with sensitivity gain from 59.2 to 64.1%.

**Table 7:** Incremental predictive value of NPAR beyond established clinical scores for three adverse outcomes.

Outcome	Model	AUC (95% CI)	Sensitivity (%)	Specificity (%)	Youden Index	*p* value^a^
Pneumonia	AIS-APS alone	0.684 (0.649–0.719)	60.3	64.9	0.252	
	AIS-APS + NPAR	0.709 (0.675–0.743)	69.0	63.3	0.323	0.0049
ICH	ASIAN score alone	0.649 (0.604–0.693)	56.6	69.2	0.258	
	ASIAN score + NPAR	0.665 (0.622–0.708)	64.6	60.6	0.252	0.11
Poor 90-day outcome	PRE score alone	0.694 (0.659–0.730)	59.2	68.3	0.276	
	PRE score + NPAR	0.701 (0.666–0.736)	64.1	64.6	0.287	0.23

a *p*-value from DeLong test for comparison of correlated ROC curves.

## Discussion

This retrospective cohort study investigated the implications of varying NPAR levels in acute ischemic stroke (AIS) patients with large vessel occlusion (LVO). Our analysis revealed significant and multifaceted differences in patient characteristics across NPAR strata. Specifically, an elevated NPAR was associated with heightened inflammatory activity (reflected in markers such as CRP, PCT, and white blood cell count), dysregulated coagulation profiles, advanced age, impaired consciousness, and a greater incidence of swallowing dysfunction. Furthermore, a high NPAR was a significant predictor of increased risks of pneumonia, intracerebral hemorrhage, and poorer 90-day functional independence, with a near-linear dose–response relationship observed for these adverse outcomes. Notably, the NPAR demonstrated consistent and stable predictive performance for these composite endpoints, suggesting its utility as a comprehensive prognostic biomarker in this patient population. NPAR’s incremental value varied across outcomes. It significantly improved pneumonia prediction, consistent with its role as an inflammatory marker. For ICH, NPAR showed a trend toward better sensitivity but non-significant AUC improvement (*p* = 0.11), possibly due to limited events (*n* = 175). For the 90-day outcome, NPAR added minimal value beyond the PRE score (*p* = 0.23), suggesting long-term prognosis is primarily determined by baseline clinical factors.

An important methodological consideration in this study is the timing of NPAR measurement. All blood samples were collected after mechanical thrombectomy (MT), following patient admission to the Emergency Intensive Care Unit (EICU). This timing is clinically relevant for several reasons. Neutrophil accumulation in the central nervous system is closely associated with neurological injury, and neutrophil extracellular traps (NETs) have been identified in thrombi from patients with acute ischemic stroke, potentially contributing to the no-reflow phenomenon through their procoagulant effects (19). As a definitive treatment for acute ischemic stroke, mechanical thrombectomy fundamentally alters the local inflammatory milieu: successful recanalization followed by reperfusion further upregulates neutrophil activity (20), thereby elevating NPAR levels. Consequently, the NPAR values measured in our study reflect not only the baseline inflammatory state associated with the ischemic stroke itself but also the acute inflammatory response to reperfusion and potential procedure-related injury.

The NPAR is increasingly recognized as a novel biomarker reflecting systemic inflammation and infection (10, 21, 22); however, it has not yet been widely adopted in clinical practice for assessing inflammatory severity. In contrast, established markers such as C-reactive protein (CRP), procalcitonin (PCT), and white blood cell count are routinely used. Our study corroborates these findings, demonstrating that patients with elevated NPAR levels also presented significantly higher levels of CRP, PCT, and leukocytes, thereby providing a foundation for future research in this area.

The associations between inflammation and subsequent reductions in platelet count and hemoglobin levels are well documented (23, 24). In line with this, we found that an elevated NPAR, indicative of a more pronounced inflammatory state, was directly correlated with lower platelet counts and hemoglobin levels—a relationship similar to that reported by Gao et al. (25). The prothrombotic effects of neutrophil activation, particularly through the release of neutrophil extracellular traps (NETs), which promote thrombosis (26–28), may explain the elevated D-dimer levels observed in our high-NPAR cohort. However, this proposed mechanism contradicts the overall coagulation profile observed in our study, which showed prolonged coagulation times. The linear relationship between NPAR and intracerebral hemorrhage indicates that risk increases incrementally with NPAR without a clear threshold, possibly reflecting consumption coagulopathy, where extensive thrombus formation depletes coagulation factors despite ongoing prothrombotic activity. Systemic inflammation may also downregulate coagulation factor synthesis while activating coagulation, creating this mixed profile that explains the coexistence of elevated D-dimer with prolonged PT/APTT/INR.

Current evidence on the relationship between the NPAR and postthrombectomy outcomes in stroke patients remains limited. Albumin is considered neuroprotective (29), whereas elevated neutrophil levels are established indicators of neurological deterioration and mortality in patients with intracerebral hemorrhage (ICH) (30–32). Similarly, Lv et al. (33) reported that the NPAR is a predictor of poor outcomes in ICH patients. Our study further revealed that high NPAR values are associated with prolonged coagulation parameters and an increased incidence of ICH, although the direct or indirect nature of this relationship remains unclear and unexplored in the literature.

As an emerging systemic inflammatory marker, the NPAR also has unique value in predicting stroke-associated pneumonia (SAP). Both Zhang et al. (22) and Tian et al. (34) confirmed associations between high NPAR levels and SAP or other infections, reporting AUC values of 0.771 (0.725–0.812) and 0.691 (0.654–0.727), respectively. Another retrospective analysis indicated that the NPAR had superior predictive value for SAP compared with the neutrophil–lymphocyte ratio (NLR) and monocyte–lymphocyte ratio (MLR) (35). Given that both ICH and pneumonia are strongly associated with increased rates of poor 90-day functional outcomes (36, 37)—a finding that is consistent with our previous research (38)—it is reasonable to conclude that the NPAR is linked to unfavorable functional independence, whether directly or indirectly.

In terms of predictive performance, established scales such as the NIHSS (39, 40), GCS (5, 34), and ASPECTS (39) are widely used in clinical practice for neurological prognosis. The comparative performance of the NPAR—a composite marker of inflammation and nutritional status—against these scales has not been previously reported. Although the NPAR demonstrated slightly lower predictive power for individual outcomes than these specialized scales did, it exhibited consistent and stable discriminatory ability across all three adverse outcomes studied. NPAR significantly improved pneumonia prediction when added to AIS-APS, enhanced sensitivity for ICH, but added minimal value for 90-day outcome beyond the PRE score. These findings suggest that the NPAR has potential as a reliable and complementary prognostic indicator in neurology.

Our findings should be interpreted in light of several constraints. First, the single-center, retrospective design may have introduced selection bias. Second, the NPAR was quantified only once—on admission to the EICU following mechanical thrombectomy—so whether temporal changes in the ratio influence patient prognosis remains untested. Additionally, due to the nature of clinical workflow, we were unable to determine the precise interval from symptom onset to blood sampling. This limits our ability to disentangle the inflammatory contributions of the initial ischemic insult from those of reperfusion injury. Finally, because all participants were Han Chinese, the external validity of the findings for other ethnicities awaits confirmation in multiethnic cohorts.

## Conclusion

In summary, higher NPAR values have a direct, linear relationship with the likelihood of pneumonia, intracerebral hemorrhage, and unfavorable 90-day recovery after acute ischemic stroke. The ratio maintains stable, albeit modest, discrimination across these disparate complications, indicating its utility as a composite proxy of disease severity. Thus, routine NPAR assessment may refine early risk stratification and inform patient prognosis in this cohort.

## Data Availability

The data supporting the findings of this observational study protocol are clinical in nature and not publicly available owing to privacy/ethical restrictions. However, the data are available from the CY upon reasonable request.
