# Supplementary material for: High neutrophil-to-albumin ratio signals severe prognosis in LVO stroke—data from an EICU cohort
Source: Front Med (Lausanne). 2026 Apr 1;13:1753227. doi: 10.3389/fmed.2026.1753227 (PMC13079661; doi:10.3389/fmed.2026.1753227)
Supplement: Supplementary file 1 [file Table_1.docx]

****Supplementary Table 1. Consistency of NPAR as a Prognostic Marker Across Key Subgroups****

| **Outcome** | **Subgroup** | **Stratum** | **OR (95% CI)** | **P value** | **P for interaction** |
| --- | --- | --- | --- | --- | --- |
| Pneumonia | Age | <70 years | 1.12 (1.07-1.18) | <0.001 | 0.685 |
|  |  | ≥70 years | 1.14 (1.08-1.19) | <0.001 |  |
|  | Sex | Female | 1.14 (1.08-1.21) | <0.001 | 0.931 |
|  |  | Male | 1.14 (1.09-1.19) | <0.001 |  |
|  | Hypertension | No | 1.18 (1.11-1.25) | <0.001 | 0.140 |
|  |  | Yes | 1.12 (1.07-1.17) | <0.001 |  |
|  | Atrial fibrillation | No | 1.12 (1.07-1.17) | <0.001 | 0.387 |
|  |  | Yes | 1.16 (1.08-1.24) | <0.001 |  |
| ****Poor 90-day outcome**** | Age | <70 years | 1.09 (1.03-1.14) | <0.001 | 0.711 |
|  |  | ≥70 years | 1.10 (1.05-1.16) | <0.001 |  |
|  | Sex | Female | 1.10 (1.04-1.17) | <0.001 | 0.955 |
|  |  | Male | 1.10 (1.05-1.15) | <0.001 |  |
|  | Hypertension | No | 1.13 (1.07-1.20) | <0.001 | 0.217 |
|  |  | Yes | 1.08 (1.03-1.14) | <0.001 |  |
|  | Atrial fibrillation | No | 1.10 (1.06-1.15) | <0.001 | 0.458 |
|  |  | Yes | 1.07 (1.00-1.14) | 0.051 |  |
| ****ICH**** | Age | <70 years | 1.07 (1.01-1.13) | 0.014 | 0.728 |
|  |  | ≥70 years | 1.06 (1.01-1.11) | 0.009 |  |
|  | Sex | Female | 1.06 (1.00-1.12) | 0.047 | 0.849 |
|  |  | Male | 1.07 (1.02-1.11) | 0.003 |  |
|  | Hypertension | No | 1.08 (1.03-1.13) | 0.002 | 0.369 |
|  |  | Yes | 1.05 (1.00-1.10) | 0.061 |  |
|  | Atrial fibrillation | No | 1.06 (1.02-1.11) | 0.008 | 0.902 |
|  |  | Yes | 1.06 (1.01-1.13) | 0.026 |  |

OR: Odds Ratio (per unit increase in NPAR); CI: Confidence Interval; ICH: intracerebral hemorrhage
